# Supplementary material for: Comprehensive Ocular and Systemic Safety Evaluation of Polysialic Acid-Decorated Immune Modulating Therapeutic Nanoparticles (PolySia-NPs) to Support Entry into First-in-Human Clinical Trials
Source: Pharmaceuticals (Basel). 2024 Apr 9;17(4):481. doi: 10.3390/ph17040481 (PMC11054942; doi:10.3390/ph17040481)
Supplement: Supplementary file 1 [file pharmaceuticals-17-00481-s001.zip › pharmaceuticals-2928703-supplementary.pdf]

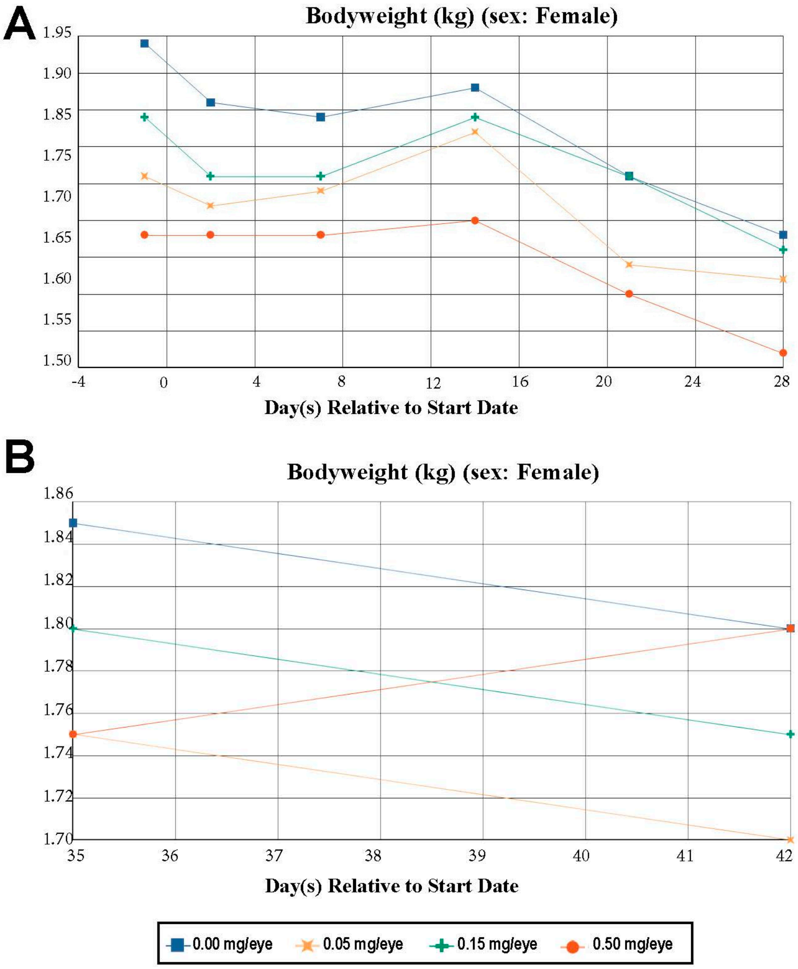

Figure S1. Bodyweight changes in female non-human primates after IVT administration of Pol-ySia-NPs recorded for 28 days (**A**) and from day 35 to day 42 (**B**).

Table S1. Assessment for abnormal clinical signs and weight changes in the micronucleus assay. Summary of clinical signs (A) and body weights (B).

Observation Type: All Types<br>Sex: Male<br>From Day -1 (Start Date) to 3 (Start Date)	Group 1, 0 mg/kg/day	Group 2, 208.75 mg/kg/day	Group 3, 417.5 mg/kg/day	Group 4, 835 mg/kg/day	Group 5, 75 mg/kg
Skin Discolored, Tail/Middle					
Days Observation Recorded	0	0	1	10	0
Number of Animals Affected	0	0	1	6	0
First to Last seen	-	-	3 - 3	1 - 3	-

A. Summary of Clinical Signs - Definitive Micronucleus Assay

Day(s) Relative to Start Date

Sex: Male		Body Weight (g) -1	Body Weight (g) 1	Body Weight (g) 2	Body Weight (g) 3	Absolute Weight Gain (g) 2 → 3	Absolute Weight Gain (g) 1 → 3
Group 1,	Mean	35.87	35.86	-	35.86	-	0.01
0	SD	1.58	1.41	-	1.88	-	0.59
mg/kg/day	N	5	5	-	5	-	5
Group 2,	Mean	35.91	35.95	-	36.27	-	0.32
208.75	SD	1.96	2.36	-	2.74	-	0.49
mg/kg/day	N	5	5	-	5	-	5
Group 3,	Mean	35.94	35.37	-	35.57	-	0.20
417.5	SD	1.81	1.82	-	1.37	-	0.62
mg/kg/day	N	5	5	-	5	-	5
Group 4,	Mean	35.78	35.40	-	35.39	-	-0.02
835	SD	2.43	2.08	-	2.42	-	0.75
mg/kg/day	N	7	7	-	7	-	7
Group 5,	Mean	35.85	-	35.55	35.56	0.02	-
75	SD	1.57	-	1.40	0.79	0.75	-
mg/kg	N	5	-	5	5	5	-

B. Summary of Body Weight Data-Definitive Micronucleus Assay
